# Timely measles vaccination in Tianjin, China: a cross-sectional study of immunization records and mothers

**DOI:** 10.1186/1471-2458-14-888

**Published:** 2014-08-29

**Authors:** Abram L Wagner, Ying Zhang, JoLynn P Montgomery, Yaxing Ding, Bradley F Carlson, Matthew L Boulton

**Affiliations:** Department of Epidemiology, School of Public Health, University of Michigan, 1415 Washington Heights, M5020 SPH II, Ann Arbor, MI 48109 USA; Division of Expanded Programs on Immunization, Tianjin Centers for Disease Control and Prevention, Tianjin, 300011 China

**Keywords:** China, Measles, Measles vaccine, Measles-mumps-rubella vaccine, Vaccination knowledge, Vaccination timeliness, Supplementary immunization activities

## Abstract

**Background:**

Measles is a highly infectious disease, and timely administration of two doses of vaccine can ensure adequate protection against measles for all ages in a population. This study aims to estimate the proportion of children aged 8 months to 6 years vaccinated on time with measles-containing vaccines (MCV) and vaccinated during the 2008 and 2010 measles supplementary immunization activities. This study also characterizes differences in mean age at vaccination and vaccination timeliness by demographic characteristics, and describes maternal knowledge of measles vaccination.

**Methods:**

Immunization records were selected from a convenience sample of immunization clinics in Tianjin, China. From the records, overall vaccination coverage and timely vaccination coverage were calculated for different demographic groups. Mothers were also interviewed at these clinics to ascertain their knowledge of measles vaccination.

**Results:**

Within the 329 immunization clinic records, child’s birth year and district of residence were found to be significant predictors of different measures of vaccine timeliness. Children born in 2009 had a lower age at MCV dose 2 administration (17.96 months) than children born in 2005 (22.00 months). Children living in Hebei, a district in the urban center of Tianjin were less likely to be vaccinated late than children living in districts further from the urban core of Tianjin. From the 31 interviews with mothers, most women believed that timely vaccination was very important and more than one dose was very necessary; most did not know whether their child needed another dose.

**Conclusions:**

When reviewing MCV coverage in China, most studies do not consider timeliness. However, this study shows that overall vaccination coverage can greatly overestimate vaccination coverage within certain segments of the population, such as young infants.

## Background

Measles is an airborne viral disease characterized by fever, cough, coryza, conjunctivitis, and a maculopapular rash, and it is treated with supportive care. While most cases recover within 3 weeks, measles can result in serious and potentially fatal complications, including diarrhea, pneumonia, blindness, and encephalitis [1, 2].

China has committed to several national and supranational measles reduction goals, including reducing measles incidence by 90% and reducing measles mortality by 95% from 1965 to 1995 [3], reducing measles incidence by 90% from 2000 to 2010, and eliminating endemic transmission of measles by 2012 [4], though this last goal was not met [5].

Morbidity and mortality from measles have decreased worldwide in recent years because of these global elimination efforts. The estimated number of measles deaths worldwide decreased from 733,000 in 2000 to 158,000 in 2011 [1]. In that time frame, however, China has seen some cyclical resurgence of measles; the government’s officially published national incidence rate increased from 5.7 cases per 100,000 persons in 2000 to 9.56 cases per 100,000 persons in 2005 [6] but then fell to 0.46 cases per 100,000 persons in 2012 [5]. The Municipality of Tianjin has had similar cycles of measles outbreaks. Between 2005 and 2008, measles incidence in Tianjin increased from 7.64 to 16.10 cases per 100,000 persons. This was followed by a period of decreasing case counts, after which the reported measles incidence in Tianjin again increased from 1.44 to 15.74 cases per 100,000 persons between 2009 and 2010 [Unpublished data, Tianjin Centers for Disease Control and Prevention (CDC), 2014].

Measles is controlled through vaccination. China has used a domestically-produced measles-containing vaccine (MCV) since 1966 [7]. Efficacy of the first dose of MCV (MCV1) is relatively low when the vaccine is administered before the child is one year of age. A review of 70 studies showed vaccine efficacy of 84.0% at 9 to 11 months of age, and 92.5% at 12 months of age or greater [8]. After a second dose of MCV, over 96% of children will have immunity to measles [9]. Therefore, a two-dose measles immunization schedule is necessary in order to ensure adequate herd immunity of this highly infectious disease within a population. A case–control study in China found that the odds of acquiring measles among those with only one MCV dose were not significantly lower than the unvaccinated children; however, those with at least two doses of MCV did have significantly lower odds of acquiring measles than the unvaccinated children [10].

Currently, the immunization schedule in Tianjin includes three MCV doses: MCV1 is a single-antigen measles vaccine given at 8 months of age, MCV2 is a measles-mumps-rubella vaccine (MMR) given at 18 to 24 months of age, and MCV3 is a second dose of MMR given at 5 years of age [11]. Most Western countries with low incidence of measles, such as the United States, follow a 2-dose MCV schedule, with MCV1 administered only at 1 year of age or older. Because of the relatively low effectiveness of MCV before 1 year of age [8], MCV administered before the child is 1 year of age in the US is not counted toward the 2-dose schedule, and 2 doses would still need to be administered to such a child after the child is 1 year of age [12]. Because of the higher incidence of measles in China, and because of the relatively high burden of measles in infants younger than 1 year of age [Unpublished data, Tianjin CDC, February 8, 2013], MCV1 is administered at less effective ages in China. However 2 doses are still given after the child is 1 year of age to ensure that adequate herd immunity can be established.

The World Health Organization (WHO) supports delivery of MCV2 through routine immunization services and through supplementary immunization activities (SIAs), which were successful in eliminating measles in the Americas in the 1990s and early 2000s [13]. “Catch-up” SIAs target all children within a particular age group and “follow-up” SIAs target children born since the previous SIA [1]. For example, Tianjin and other provinces in China implemented a catch-up SIA in 2008 which targeted all children aged 8 months to 14 years of age, regardless of prior vaccination status. The SIAs delivered 1.3 million doses in Tianjin and 46.5 million doses total throughout China. The 2010 follow-up SIA targeted all children aged 8 months to 4 years in Tianjin, regardless of vaccination status; 450,000 doses were delivered in Tianjin, and 102.3 million doses were delivered in China [14].

Maternal knowledge of a certain vaccine may affect vaccine timeliness. One study of caregivers of infants in China showed that infants whose caregivers scored lower on vaccine knowledge had higher prevalence of delayed hepatitis B immunization at 12 months of age compared to caregivers with the highest knowledge score [15]. Moreover, parents aware of the necessity and importance of timely vaccination are more likely to vaccinate their child on time [16, 17].

The degree of delay in measles vaccinations and knowledge regarding measles vaccinations in Tianjin, China, are of particular interest because of the difficulties in reducing measles incidence in the elimination phase of disease control. This study aims to estimate the proportion of children aged 8 months to 6 years who were vaccinated on time with MCVs and vaccinated during the 2008 and 2010 SIAs. This study also characterizes differences in mean age at vaccination and describes maternal knowledge of measles vaccination.

## Methods

From June to August 2011, 4 immunization clinics were visited in Tianjin, a municipality in northeastern China. Tianjin had 12.9 million residents in its 2010 census; 4.3 million lived in the heavily urbanized central districts, 5.0 million lived in suburb districts, and 3.6 million lived in rural districts [18]. Urban areas in Chinese cities are generally wealthier than non-urban areas [19], and generally have been developing faster compared to non-urban areas [20].

Immunization clinics serve specific geographical regions, and all children in Tianjin receive measles vaccinations at these government-funded clinics. Each district has approximately 10 to 20 immunization clinics. Children generally do not attend multiple clinics over the course of their childhood unless their family moves to a different area, in which case their record would be transferred to that new area.

We selected four clinics, one from each of the following districts: Hebei (urban), Binhai (suburban), Ninghe (rural), and Wuqing (rural). These clinics were selected because they were accessible and because they represented the diversity of different residential environments (i.e. urban, suburban, and rural) within Tianjin. Two rural clinics were chosen to increase the sample size from that setting. At each clinic a review of a random sample of immunization records was conducted. Additionally, at the same clinics where records were sampled, a sample of mothers accompanying their children for immunization appointments was selected, and the mothers were interviewed individually about their knowledge of measles vaccination and their child’s immunization history. Although this immunization history was similar to the information collected as part of the record review, data from the mother interviews were not added to the record review because of the different sampling techniques.

### Record review

At each of these clinics, a sample of vaccination records was reviewed for MCV information. Paper records were first ordered by birth year, and the number of children in each birth year was tallied. Within each birth year between 2005 and 2010, one-tenth of the records were selected using a random numbers table. The study sample size chosen was sufficient to evaluate the significance of at least one month difference in mean age at vaccination across birth years, assuming a standard deviation of one month. District of residence, birthdate, sex, dates of MCV administration, and MCV type (routine measles vaccine, routine MMR, SIA measles vaccine) were abstracted from each record onto a standardized data collection form. SIA administration information was recorded in a special file and in the child’s vaccine booklet at the time of the SIA, and later transferred to the child’s permanent immunization record in the clinic.

The following variables were derived from the vaccination records: child’s age (date of data collection minus date of birth), child’s age at each vaccination (date of vaccination minus date of birth), timely vaccination, and SIA eligibility. Vaccinations were considered timely if administration occurred within 8 months for MCV1, before 2 years for MCV2, and before 5 years 1 month for MCV3. Eligibility for SIAs was defined as 8 months of age and older for SIA 2008 or aged 8 months to 4 years for SIA 2010. For both SIAs, subjects were designated ineligible if vaccinated with an MCV in the 30 days before the SIA. Although the SIAs were carried out in Ninghe, records from this district did not include SIA vaccinations, so it was not possible to calculate SIA for Ninghe district.

For routine immunizations, the mean ages at the three MCV doses were calculated and stratified by child’s resident district, birth year, and sex. A non-parametric Wilcoxon rank-sum test assessed significance of differences in mean age at vaccination between the sexes, and the Kruskall-Wallis test evaluated the significance in mean age at vaccination across birth years. The proportions of subjects vaccinated in the 2008 and 2010 SIAs were also calculated and stratified by district, birth year, and sex; significance was assessed through a Fisher’s exact test.

### Mother interviews

A small sample of mothers was chosen in order to perform a pilot study on the diversity of maternal knowledge and attitudes towards measles vaccinations. All women who came to the clinic for a vaccination appointment on study days during June to August 2011 were invited to participate in the study. If they were a mother of a child aged 8 months to 6 years, they were interviewed after giving verbal consent. The mothers were asked about their age, their highest level of completed education, and their homes’ distance from the clinic in minutes travel time. Knowledge about measles vaccination was assessed on a three-point scale by asking mothers how important timely vaccination was to protect their children from getting measles (not important, somewhat important, or very important), how necessary more than one dose was to protect their children from getting measles (not necessary, somewhat necessary, or very necessary), and whether their child needed another dose (yes or no). The time required to travel to the clinic from the client’s home was dichotomized to 10 minutes or less and more than 10 minutes.

The record review sample size calculation used nQuery (Statistical Solutions Ltd, Boston, MA). The data collection forms for both the record review and mother interviews were entered into databases in OpenClinica (OpenClinica, LLC, Waltham, MA) with double data entry. All analyses were performed with SAS, v. 9.2 (SAS Institute, Cary, NC).

The study was approved by the institutional review board at the University of Michigan, Ann Arbor, and the ethical review committee at the Tianjin Centers for Disease Control and Prevention in Tianjin, China, determined that the project was exempt from ethical review.

## Results

### Record review

Of the 329 children whose records were reviewed, there were approximately equal numbers of each sex, 258 (78.4%) came from Hebei district, and all birth year cohorts had at least 40 children (Table 1). Administration of MCV1 was recorded for 324 children, the average age at MCV1 was 8.46 months, and most children (87.5%) were vaccinated on time. For MCV2, 265 children had a record of administration, the average age at MCV2 was 20.54 months, and a large majority of children (93.1%) were vaccinated on time. MCV3 was recorded for 67 children, the average age at MCV3 was 5.04 years, and slightly more than three-fourths of children who were eligible for vaccination were vaccinated on time (Table 1).

**Table 1:** **Mean age at vaccination and prevalence of on-time vaccination among children in Tianjin, China, summer 2011**

	MCV dose 1 ^a^		MCV dose 2 ^b^		MCV dose 3 ^c^		
	Mean age in months at vaccination (SD)	Number vaccinated on-time (%)	Mean age in months at vaccination (SD)	Number vaccinated on-time (%)	Mean age in years at vaccination (SD)	Number vaccinated on-time (%)	Total number
**Overall**	8.46 (0.78)	288 (87.5%)	20.54 (2.94)	246 (93.1%)	5.04 (0.14)	52 (76.5%)	329
**Sex**							
Female	8.37 (0.54)	156 (90.2%)	20.68 (2.86)	131 (94.2%)	5.05 (0.17)	30 (73.2%)	173
Male	8.57 (0.97)	132 (84.6%)	20.37 (3.02)	115 (91.3%)	5.03 (0.03)	22 (81.5%)	156
**District** ^**d**^							
Binhai	8.82 (0.91)	30 (63.8%)	22.48 (5.40)	24 (68.6%)	5.07 (0.09)	3 (27.3%)	47
Hebei	8.38 (0.70)	239 (92.6%)	20.13 (1.82)	207 (98.6%)	5.04 (0.09)	46 (92.0%)	258
Ninghe	8.84 (1.48)	9 (75.0%)	20.56 (2.14)	9 (81.8%)	5.01 (0.00)	1 (33.3%)	12
Wuqing	8.55 (0.60)	10 (83.3%)	23.36 (7.07)	6 (66.7%)	5.07 (0.57)	2 (50.0%)	12
**Birth year**							
2005	8.59 (0.97)	37 (90.2%)	22.00 (4.71)	36 (87.8%)	5.07 (0.15)	30 (73.2%)	41
2006	8.42 (0.37)	50 (94.3%)	21.76 (3.30)	49 (92.5%)	4.99 (0.11)	22 (81.5%)	53
2007	8.50 (0.59)	48 (85.7%)	20.61 (1.49)	53 (94.6%)	-	-	56
2008	8.35 (0.66)	50 (94.3%)	20.10 (1.86)	50 (94.3%)	-	-	53
2009	8.59 (1.12)	47 (77.1%)	18.83 (1.45)	54 (93.1%)	-	-	61
2010	8.37 (0.75)	56 (86.2%)	17.96 (0.03)	4 (100.0%)	-	-	65

MCV, measles-containing vaccine.

SD, standard deviation.

^a^329 children were eligible for MCV1. Children 8 months of age or older were included. On-time vaccination is defined as administration before the infant is 9 months of age.

^b^265 children were eligible for MCV2. Children 18 months of age or older were included. On-time vaccination is defined as administration before the child is 2 years of age.

^c^68 children were eligible for MCV3. Children 5 years of age or older were included. On-time vaccination is defined as administration before the child is 5 years 1 month of age.

^d^Only one clinic per district was selected.

No significant differences between sexes were found for mean age at vaccination or vaccination timeliness. Age at MCV1 administration (P = 0.0003), MCV2 administration (P < 0.0001), and MCV3 administration (P = 0.0019) differed by birth year. Compared with children from other districts, children living in Hebei district had the highest prevalence of timely vaccination (P < 0.0001 for all three MCVs).

Of 136 children eligible for the 2008 SIA, 121 (89.0%) participated. Of 197 children eligible for the 2010 SIA, 123 (62.4%) participated. For both SIAs, variation in participation by sex and across district of residence was not statistically significant (Table 2).

**Table 2:** **Participation in measles SIAs among children in Tianjin, China, summer 2011**

	Participated in 2008 SIA ^a^ (%)	Participated in 2010 SIA ^b^ (%)
**Overall**	121 (89.0%)	123 (62.4%)
**Sex**		
Female	66 (88.6%)	64 (63.4%)
Male	55 (88.2%)	59 (61.5%)
**District** ^**c**^		
Binhai	4 (44.4%)	7 (63.6%)
Hebei	116 (95.9%)	110 (61.5%)
Wuqing	5 (83.3%)	6 (85.7%)
**Birth year**		
2005	31 (88.6%)	3 (37.5%)
2006	39 (83.0%)	17 (36.2%)
2007	45 (93.8%)	22 (44.9%)
2008	6 (100.0%)	40 (87.0%)
2009	-	41 (95.4%)
2010	-	0

SIA, supplementary immunization activity.

^a^136 children were eligible for the 2008 SIA (41.3% of the study population). Children were eligible if they had no measles-containing vaccine in the previous month and if they were 8 months of age or older at the time of the SIA.

^b^197 children were eligible for the 2010 SIA (59.9% of the study population). Children were eligible if they had no measles-containing vaccine in the previous month and if they were 8 months to 4 years of age at the time of the SIA.

^c^Only one clinic per district was selected.

### Mother interviews

A total of 42 women were approached to be interviewed in the immunization clinics. Eight were ineligible to participate in the study because their infants were younger than 8 months of age. Three mothers were eligible but chose not to participate in the study. Table 3 shows the demographic characteristics of the participants. Of the 31 total participants, 23 (74.2%) were from Hebei district and the rest were from Binhai, Ninghe, or Wuqing districts. The mothers were evenly divided between those who had completed college (15, 48.4%) and those who had lower levels of education. Most women (22, 75.9%) lived ≤10 minutes from the clinic.

**Table 3:** **Demographic characteristics and attitudes toward measles vaccine in a sample of mothers in Tianjin, China, summer 2011**

	Number	%
**Overall**	31	100
**Child’s sex**		
Female	17	54.8
Male	14	45.2
**District**		
Binhai	4	12.9
Hebei	23	74.2
Ninghe	2	6.5
Wuqing	2	6.5
**Mother’s age** ^**a**^		
< 28 years	7	23.3
28 to <30 years	4	13.3
30 to <32 years	10	33.3
≥ 32 years	9	30.0
**Mother’s highest education**		
Middle school	8	25.8
High school	4	12.9
Vocational education	3	9.7
Junior college	1	3.2
College or above	15	48.4
**Minutes from clinic** ^**a**^		
≤ 10 minutes	22	75.9
> 10 minutes	7	24.1
**Importance of on-time measles vaccination**		
Not important	0	0
Somewhat important	2	6.5
Very important	28	90.3
I do not know	1	3.2
**Necessity of more than one dose of measles vaccine**		
Not necessary	0	0
Somewhat necessary	11	35.5
Very necessary	18	58.1
I do not know	2	6.5
**Child needs another dose of measles vaccine**		
Yes	6	19.4
No	4	12.9
I do not know	21	67.7

^a^Data were missing for 1 mother about age and for 2 mothers about distance from the clinic.

Mothers’ attitude towards and knowledge of measles vaccines was assessed through three questions. A large majority of mothers (29, 90.3%) believed that timely vaccination was “very important.” A slight majority (18, 58.1%) believed that more than one dose of MCV was “very necessary” to protect their child. About two-thirds (67.7%) of mothers did not know whether their child needed another dose or not.

Mother’s attitude towards measles vaccination was not significantly associated with most other variables. The number of women indicating that more than one MCV dose was “very necessary” to protect their child did not vary across district (P = 0.12), child’s birth year (P = 0.62), child’s sex (P = 0.92), mother’s highest education level (P = 0.34), minutes from clinic (P = 0.60), or vaccine timeliness (P = 0.37). However, the number of women indicating that more than one dose of MCV was “very necessary” did differ by mother’s age group (P = 0.0181): only 14.3% of mothers younger than 28 years thought more than one dose to be very necessary, whereas 88.9% of mothers greater than or equal to 32 years of age thought this.

## Discussion

Measles vaccination is an important indicator of progress toward the fourth Millennium Development Goal of reducing the under-5 mortality rate [1, 4]. However, for immunization programs to successfully combat measles, vaccination of children needs to be timely and two doses are required [9, 10]. We found levels of timely MCV1 administration to be lower than 90% in children living in Tianjin, China. Given that measles vaccine is only about 84.0% effective at this age [8], the establishment of herd immunity may be delayed in this population and more infants may be at risk of acquiring measles than indicated by an MCV1 coverage estimate that does not consider timeliness. Moreover, timely vaccination of MCV2 varied across time and geography, indicating that measles susceptibility in Tianjin varies by residence and by birth cohort.

When reviewing MCV coverage in China, studies typically do not consider timeliness [21]. However, this study shows that only looking at overall vaccination coverage, and not timeliness, can greatly overestimate vaccination coverage within certain segments of the population, like young infants. For example, in this study, 98.5% of the children had been vaccinated with MCV1 but only 87.5% had been vaccinated on-time. These figures are similar to a 2011 internal report of the Tianjin CDC, which showed that 99.0% of surveyed children had an MCV1 record, but only 85.9% had been vaccinated on time [Personal Communication, Tianjin CDC, 2011]. Considering that infants are at high risk for acquiring measles, timely vaccination is important. Reporting vaccination coverage, instead of timely vaccination coverage, may not provide the true picture of protection against measles in the population of young infants.

Of the 136 children eligible for the 2008 SIA in this study, only 88.4% participated; 62.4% of the 197 eligible children participated in the 2010 SIA. These are lower estimates than those previously described. For example, a report from the Tianjin Beichen district CDC of 71,581 children between 8 months and 14 years of age with electronic vaccination records found a very high (99.8%) level of participation in the 2008 SIA [22]. The age range for the study in Beichen district had a higher bound than in our study (7 years of age). The study in Beichen district did not report SIA participation by age, but if older ages had higher participation, then the findings in our two studies may not be directly comparable. Our study may also have underestimated SIA participation because children should not receive an MCV within 28 days of another live vaccine, such as oral polio or varicella if not administered simultaneously [23]. However, only information on MCV administration was collected for this study; children receiving oral polio or varicella could have also been ineligible but would have still been considered eligible in our calculations. Low SIA estimates may also result from a child’s SIA record not being transferred to the clinic records; this was considered a low possibility, however, because the original record was recorded both in the child’s vaccine booklet and in a special file. Clinics used both of these documents to update children’s records. Lastly, the low estimate of SIA participation in this study could represent a reduced willingness of Chinese parents to participate in the SIAs, the effect of which could vary across districts. These SIAs targeted children regardless of their vaccination status, and future research could investigate potential parental fatigue from receiving multiple doses of the same vaccine through both routine immunization services and SIAs.

From the 31 interviews with mothers in this study, most women believed that timely vaccination was very important and more than one dose was very necessary, but most did not know whether their child needed another dose. Because of the small sample size, we did not relate maternal knowledge to vaccination outcomes in the child. Other studies, like that by Richards and Sheridan in Australia, have found that maternal knowledge of the vaccine was important for predicting vaccine timeliness [16]. In a survey of migrants and migrant children in Zhejiang province, China, maternal attitudes and knowledge, including perception of vaccine necessity and knowledge of the immunization schedule, were associated with measles vaccination and timeliness of measles vaccination [24].

In previous studies, factors such as mother’s education level and age have been related to mother’s knowledge of vaccination. Although we did not see a significant difference across maternal age, one study in Gansu, China [15] did show mother’s education level to be related to various child vaccine statuses. Necessity of more than one dose of MCV did vary by mother’s age in our study; older mothers were more aware of the necessity of more than one dose of MCV. Studies in the United States [25] and Kenya [26] have similarly found maternal age to affect child vaccination status. This finding could be a result of mothers being more experienced with the vaccination programs from previous children, though this is likely to be less of a factor in China because of its One Child Policy.

Another limitation to the study was that most participants came from Hebei (79.12% of records and 74.19% of mothers), a wealthier, urbanized district in Tianjin. The high proportion of participants from Hebei could lead to results unrepresentative of Tianjin as a whole. In addition, because we purposely chose each clinic, they may not be representative of the urban-suburban division in Tianjin. Instead, they reflect some of the diversity seen in vaccination outcomes throughout the city. Because we only included the immunization history from children who had attended an immunization clinic and only interviewed mothers who brought their children to immunization clinics, the possibility of selection bias exists. We may have underestimated vaccination outcomes in the record review and our study’s mothers may have been more knowledgeable than mothers who did not attend the immunization clinic. Attendance at immunization clinics in China is not well documented, but one study among parents who recently moved from rural areas to Beijing found that 11.9% of them had not yet brought their child to an area immunization clinic [27]. Future studies with a larger sample size will be better able to analyze the relationship between attitudes towards vaccination and vaccination completeness or timeliness in China, and these studies could also include provider-level variables such as availability of vaccines at clinics.

## Conclusion

Measles is a difficult-to-control infectious disease because of its high degree of communicability, resulting in the necessity of a two-dose immunization schedule. China has attempted to deliver this second dose through the routine immunization program augmented by supplementary immunization activities. This study found decreases in mean age of vaccination, especially for MCV2, over time, but important differences in vaccination timeliness across different geographical regions mean that susceptible pockets of population may still exist despite overall high coverage of MCV. Reporting of vaccination coverage should include statistics on whether administration of the dose was on time, especially for countries like China that are trying to eliminate measles. This information is particularly important for highly infectious diseases like measles, pertussis, and varicella, which require a high threshold of timely vaccination to stop the spread of disease in young infants.

## Abbreviations

CDC: Centers for disease control and prevention
MCV: Measles-containing vaccine
MMR: Measles-mumps-rubella vaccine
SIA: Supplementary immunization activity
WHO: World Health Organization.
